# CD4^+^ T cells in ischemic stroke: effects and therapeutic targets

**DOI:** 10.3389/fimmu.2025.1512634

**Published:** 2025-04-25

**Authors:** Zhengqi Lian, Ying Luo, Yina Li, Yikun Gao, Xiaoxing Xiong, Lijuan Gu

**Affiliations:** ^1^ Central Laboratory, Renmin Hospital of Wuhan University, Wuhan, China; ^2^ Department of Neurology, Renmin Hospital of Wuhan University, Wuhan, China; ^3^ Department of Neurosurgery, Renmin Hospital of Wuhan University, Wuhan, China

**Keywords:** ischemic stroke, CD4^+^ T cells, Th17 cells, Treg cells, inflammatory responses, therapeutic target

## Abstract

Ischemic stroke (IS) is a significant contributor to disability and death worldwide, with limited treatments beyond early intervention. The importance of CD4^+^ T cells in the advancement of IS has been highlighted by recent studies, providing new insights into immunomodulatory strategies. This review describes the spatiotemporal dynamics of CD4^+^ T cells and their subsets at different stages of IS. The signaling pathways activated by IS regulate the distribution of CD4^+^ T cells and their subsets, which further influences the inflammatory response and disease progression. In the acute and subacute stages, CD4^+^ T cells exacerbate neuronal damage. In contrast, CD4^+^ T cells, which are predominantly composed of Treg cells (Tregs), promote tissue repair and neurological recovery in the chronic stage. In light of recent findings that challenge traditional views, we analyze the underlying mechanisms and potential explanations for these discrepancies. In addition, we summarize the potential of targeting CD4^+^ T cells as a therapeutic strategy for IS. Although no drugs specifically targeting CD4^+^ T cells have been developed, certain drugs that modulate CD4^+^ T cells show potential for IS treatment. Moreover, multitarget drugs integrated with nanomaterials are currently undergoing preclinical investigation. We further explore the challenges in the clinical translation of CD4^+^ T-cell-targeted therapies and discuss potential strategies to address these challenges. In conclusion, a deeper comprehension of the complex effects of CD4^+^ T cells and their subsets on IS will contribute to disease management and drug development, thereby improving the quality of life for IS patients.

## 1 Introduction

Stroke has become the second greatest cause of disability and death globally, with ischemic stroke (IS) accounting for 87% of all stroke cases (1). Early revascularization has a narrow therapeutic window, is not helpful for late functional recovery, and can cause secondary reperfusion-induced brain injury (2). Immunomodulatory strategies are important, as neuronal damage after IS initially results from sterile inflammation caused by the damage-associated molecular patterns (DAMPs) release (3).

The involvement of CD4^+^ T cells is essential in this process. Due to the varying effects of these cells on different stages of IS, the acute and subacute phases are collectively classified as the early stage, whereas the chronic phase is referred to as the late stage (the acute phase: 0–7 days after IS; the subacute phase: 7 days–3 months after IS; and the chronic phase: from 3 months onward) (4). In the acute phase, DAMPs activate immune cells with a proinflammatory phenotype and induce an inflammatory cascade (5). The damaged blood‒brain barrier (BBB) allows CD4^+^ T cells to infiltrate the lesion site within 24 hours after IS (6). These cells present typical features of innate immune cells at this stage (7). In the subacute phase, CD4^+^ T cells continue to infiltrate the infarct region in a time-dependent manner, further exacerbating neuronal damage (8). However, in the chronic phase, although CD4^+^ T cells still infiltrate the brain, the inflammatory response gradually diminishes, which promotes tissue repair and regeneration (9). However, different studies have reached conflicting conclusions concerning the effects of CD4^+^ T cells on IS. These contradictions were clarified by the discovery of CD4^+^ T-cell subsets, which have varying effects on different stages of IS (10, 11). Given the complexity of the mechanisms of different CD4^+^ T-cell subsets in IS, a comprehensive analysis of their effects on the immune response after IS is essential for understanding the pathological process of IS and identifying potential therapeutic targets.

Immunosuppressive therapy for IS is necessary during the acute and subacute phases due to brain inflammation, whereas the periphery is in a state of immunosuppression because massive peripheral immune cells migrate to the brain (12). Therefore, immunosuppressive therapy further exacerbates peripheral immunosuppression, which leads to severe infections in patients with acute ischemic stroke (AIS). The central and peripheral immune states are also reversed in the chronic phase. Therefore, neither immunosuppression nor immune activation alone can involve both central and peripheral treatment after IS. CD4^+^ T cells and their subsets are potential immunomodulatory targets for IS (13–15). Owing to the spatial and temporal distribution of CD4^+^ T cells, targeting these cells can flexibly regulate central and peripheral immune responses. In addition, the selective upregulation of anti-inflammatory subsets and downregulation of proinflammatory subsets is possible according to the effects of different CD4^+^ T-cell subsets. This approach accounts for the different characteristics of central and peripheral immune responses at different stages of IS, thereby avoiding unnecessary immunosuppression or excessive inflammation. Moreover, CD4^+^ T-cell-targeted therapy in the chronic phase extends the therapeutic window for IS patients, which offers potential benefits for patients who are ineligible for revascularization therapy. Therefore, targeting CD4^+^ T cells and their subsets has significant clinical value.

This review analyzes the temporal distribution and functional characteristics of different CD4^+^ T-cell subsets involved in the poststroke immune response and summarizes clinical agents that target CD4^+^ T cells for IS treatment. We aim to provide new insights and potential directions for immunotherapy for IS.

## 2 Subsets and functions of CD4^+^ T cells

The differentiation of CD4^+^ T cells begins with classical costimulatory signals necessary for their activation (16, 17). After the invasion of different antigens into an organism, antigen-presenting cells (APCs) facilitate the formation of adaptive tissue microenvironments. The binding of MHC II molecules on APCs and T-cell receptors (TCRs) on CD4^+^ T cells provides the first signal, and stimulatory or inhibitory costimulatory molecules on APCs deliver the second signal by binding to CD28 receptors on CD4^+^ T cells. These costimulatory molecules modulate key transcription factors within CD4^+^ T cells, thereby guiding their differentiation into distinct subsets. The tissue microenvironment induced by APCs simultaneously contains a diverse array of cytokines. These cytokines regulate gene expression in CD4^+^ T cells to form specific functional effector subsets.

**Figure 1:**
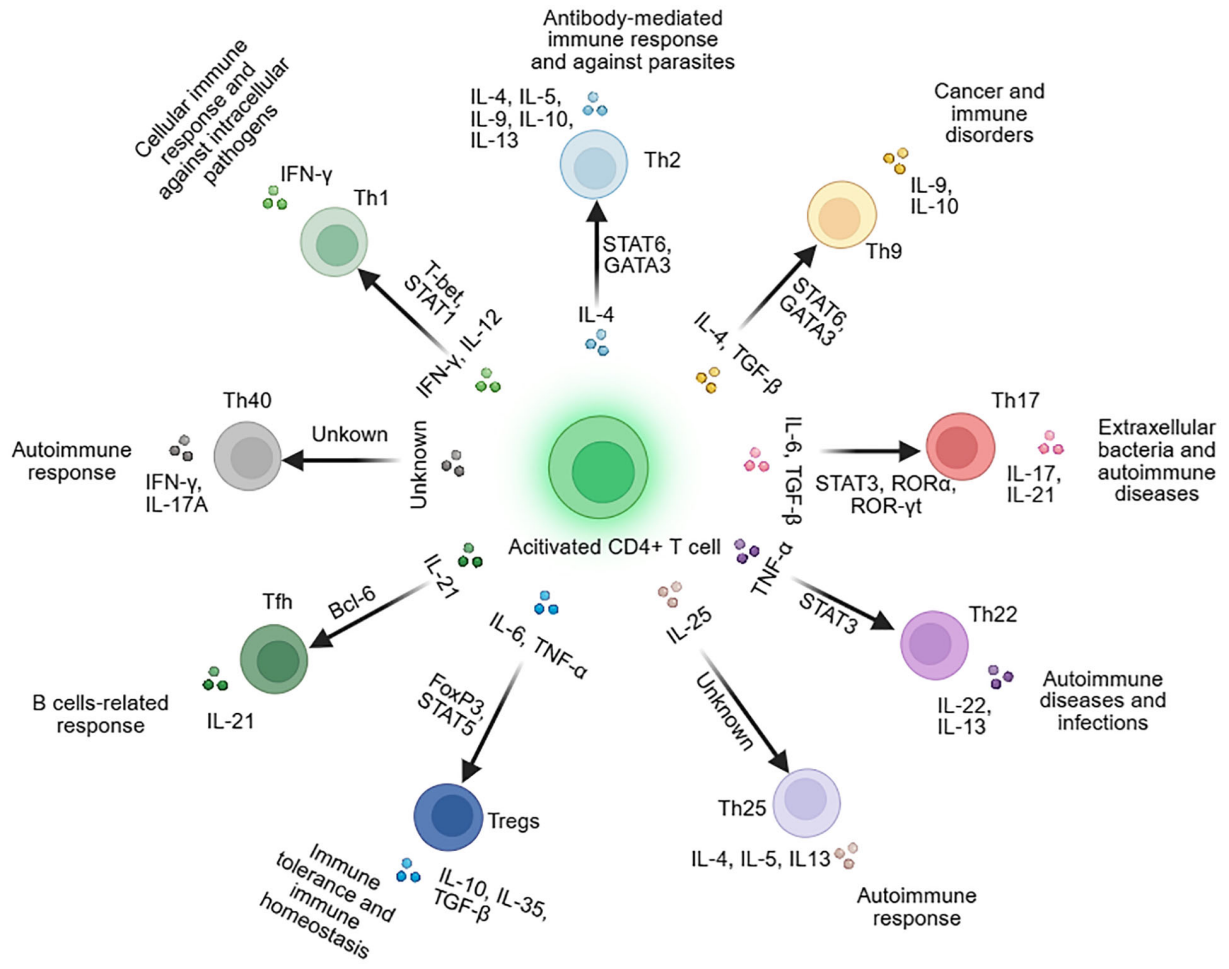
Subsets and functions of CD4^+^ T cells. (**Figures 1**-**4**: Created with BioRender.com).

The antigen-driven molecular expression of APCs and the formation of distinct cytokine environments determine the differentiation of CD4^+^ T-cell subsets, which ultimately enables adaptive immune responses to specific antigenic challenges (18). Currently, researchers have identified nine CD4^+^ T-cell subsets. Their differentiation mechanisms and functions are specifically illustrated in **Figure 1**.

## 3 Spatiotemporal dynamics of CD4^+^ T cells and their subsets in IS

**Figure 2:**
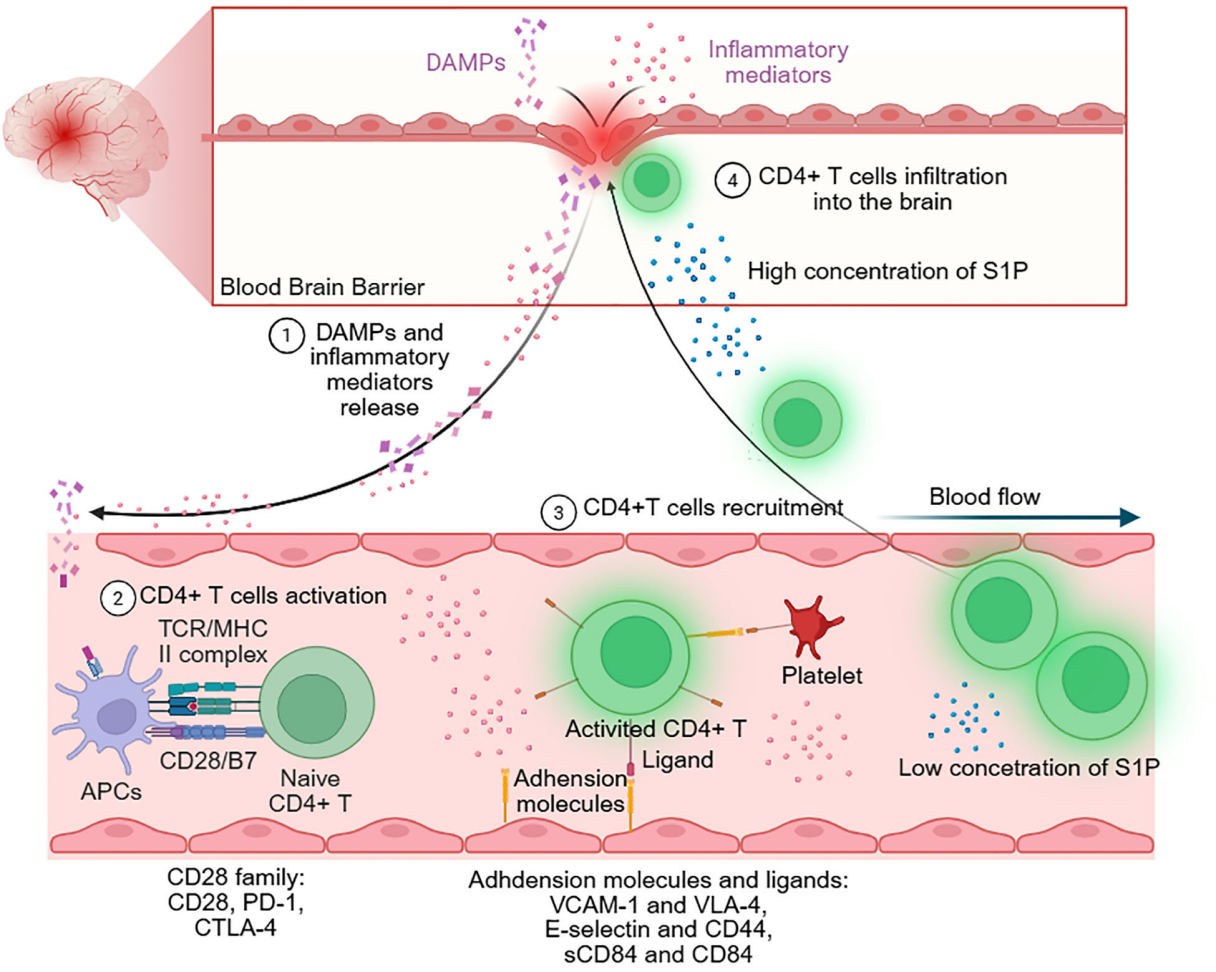
CD4^+^ T-cell response after ischemic stroke (IS). Following IS, DAMPs release from the injured blood-brain barrier (BBB) to the periphery and are presented by antigen-presenting cells (APCs). APCs activate circulating CD4^+^ T cells by the binding of MHC II and TCR, which is the first stimulating signal of CD4^+^ T cell activation. The second stimulating signal of CD4^+^ T cell activation is the binding of CD28 and B7. CD28 family includes CD28, PD-1 and CTLA-4, in which PD-1 and CTLA-4 play the inhibitory role in CD4^+^ T cell activation. After being activated, CD4^+^ T cells are recruited into the brain. The expression of adhesion molecules such as VCAM-1 and E-selectin promote the recruitment and infiltration of CD4^+^ T cells. Meanwhile, inflammatory mediators and S1P also induce CD4^+^ T cells to flow into the brain.

Following IS, the initial activation and proliferation of CD4^+^ T cells are antigen-independent. CD4^+^ T cells are activated through a classical antigen-dependent response only in the late stage of IS (7). Many molecules, such as chemokines (19) and the lipid molecule sphingosine-1-phosphate (S1P) (20), signal the recruitment of CD4^+^ T cells. CD4^+^ T cells subsequently migrate through interactions with adhesion molecules on the vascular endothelial cell surface and eventually infiltrate the ischemic infarct region through the disrupted BBB (21). Recent studies have reported that the choroid plexus and meninges might be other routes for CD4^+^ T-cell entry following IS (22) (**Figure 2**).

CD4^+^ T cells rapidly decrease in number in the circulation and infiltrate the brain within 24 hours following IS (6). Within the first week, the brain contains few CD4+ T cells, and their numbers increase significantly by day 7 and persist for up to 30 days. In the early stage, Th1, Th17, and follicular helper T cells (Tfh) cells are the predominant CD4^+^ T cells (23). The number of these cells decreases by day 7 and gradually increases in the late stage. In contrast, Th2 cells exhibit the opposite pattern. Treg cells (Tregs) are rarely observed in the brain in the early stage (24). Their numbers increases on day 7 and persists throughout the late stage of IS.

These findings indicate that CD4+ T-cell subsets enter the brain at different stages of IS, with the process being affected by different molecules (**Table 1**). In early IS, microRNA-494-3p induces Th1 differentiation through the upregulation of gene transcription (25). Mucosa-associated lymphoid tissue lymphoma translocation protein 1 (MALT1) and Interferon gamma (IFN-γ) promote Th1 and Th17 cell proliferation by increasing the levels of differentiation-related cytokines (26, 27). The regulation of cell apoptosis by FasL (28) and cell metabolism by acetyl coenzyme A carboxylase 1 (ACC1) (29) also increases the proportion of Th17/Treg ratio. Additionally, CXCL13 expression increases the recruitment of Tfh cells into the infarct region (30). In the late stage, IL-6 and IL-33 promote Th2/Treg differentiation. They inhibit Th1/Th17 differentiation by modulating the secretion of the corresponding cytokines at the same time (31, 32).

**Table 1:** The modulation of CD4^+^ T-cell differentiation after IS.

Molecule name	Molecule type	Effect	Mechanism	Author	Year
MicroRNA-494	MiRNA	Th1 shift	Promoting the STAT4 transcription	Zhao et al. (25)	2020
MALT1	paracaspase	Th1 and Th17 shift	Activating NF-κB signaling pathway	Chen et al. (26)	2021
IFN-γ	chemokine	Promoting Th1 proliferation and chemotaxis to the injured site	Serving as related differentiation cytokines	Seifert et al. (27)	2014
CXCL13	chemokine	Increasing Tfh recruitment	Promoting cell recruitment	Rayasam et al. (30)	2022
FasL	Death receptor ligand	Promoting Th17 while suppressing Tregs	Regulating cell apoptosis	Zhao et al. (28)	2018
IL-6	cytokine	Promoting Th2/Tregs; inhibiting Th1	Affecting corresponding cytokines secretion	Yao et al. (31)	2019
IL-33	cytokine	Promoting Th2/Tregs; inhibiting Th1/Th17	Affecting corresponding cytokines secretion	Guo et al. (32)	2023

The spatiotemporal distribution of CD4^+^ T cells and their subsets after IS is closely linked to their effects on disease progression. The inflammatory microenvironment induced by IS regulates the migration and functional states of these cells over time, thereby shaping their immunomodulatory effects during different stages (5). These dynamic changes suggest that CD4^+^ T-cell subsets infiltrating the brain during early IS exacerbate cerebral damage, whereas those present in late IS facilitate functional recovery (**Table 2**). However, recent findings challenge the conventional understanding. Th17 cells, which are proinflammatory immune cells, might promote neurogenesis and angiogenesis in the late stage of middle cerebral artery occlusion (MCAO) (33, 34). Although numerous studies have reported that Tregs reduce the infarct volume by suppressing inflammatory responses after MCAO (35), Tregs might also contribute to cerebrovascular endothelial dysfunction in the early stage of IS (36). Next, we discuss in detail the roles and mechanisms of CD4^+^ T cells and their subsets.

**Table 2:** The effects of CD4^+^ T cells and their subsets on IS at different stages.

Phases of stroke	Types of CD4^+^ T cells	Effects on stroke	Mechanisms
Acute phase	CD4^+^ T cells	Detrimental	Promoting neuronal death
	Th1 cells	Detrimental	Disrupting the BBB; Promoting inflammatory response
	Th2 cells	Protective	Promoting neuronal survival
	Th17 cells	Detrimental	Impairing the BBB integrity; Inducing neuronal death
	Tregs	Paradoxical	Inhibiting neuroinflammation; Increasing thrombo-inflammatory
	Th22 cells	Protective	Inhibiting neuronal apoptosis; Suppressing oxidative stress; Reducing inflammatory response
	Tfh cells	Detrimental	Promoting neuronal apoptosis
	CD4+CD28- T cells	Detrimental	Increasing proinflammatory cytokines release
Subacute phase	CD4^+^ T cells	Detrimental	Promoting neuronal death
	Tregs	Protective	Inhibiting proinflammatory response; Promoting the BBB repair
Chronic phase	CD4^+^ T cells	Detrimental	Increasing neurological deficits
	Th1 cells	Protective	Promoting neuronal survival
	Th2 cells	Protective	Promoting brain repair
	Th17 cells	Unsure	Regulating neurogenesis and angiogenesis
	Tregs	Protective	Promoting brain repair

## 4 Effects of CD4^+^ T cells on different stages of IS

### 4.1 CD4^+^ T cells promote neuronal death in early IS

**Figure 3:**
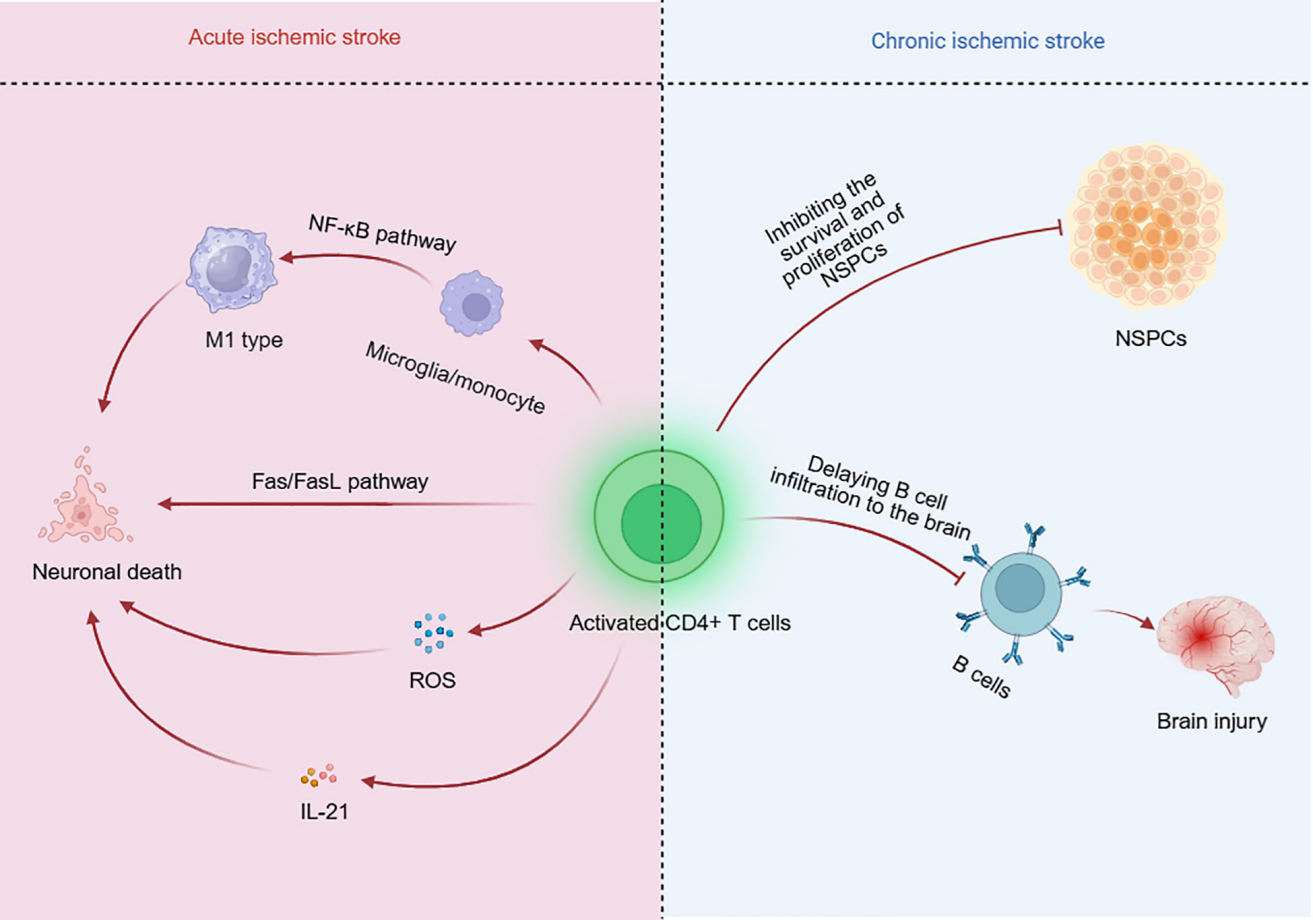
Effects of CD4^+^ T cells on ischemic stroke (IS). In the early stage of IS, CD4^+^ T cells induced neuronal death in multiple manners. These cells directly kill neurons by activating Fas/FasL signaling pathway. In addition, CD4^+^ T cells promote M1 phenotype polarization by NF-κB pathway and then induce neuronal death. Moreover, the secretion of IL-21 and reactive oxygen species (ROS) increase the neuronal injury. In the late stage of IS, CD4^+^ T cells reduce the survival and proliferation of neural stem/progenitor cells (NSPCs). These cells also induce delayed B cells to infiltrate into the infarct region, which alleviates the poststroke cognitive impairment (PSCI).

The massive cell death caused by the inflammatory response during the early stage of IS leads to severe brain damage. CD4^+^ T cells induce neuronal death through cell–cell interactions and interleukin secretion (**Figure 3**).

Following IS, the expression of FasL increases on CD4^+^ T cells. The interaction between FasL on CD4^+^ T cells and Fas on neurons directly kills neurons by activating apoptosis-related signaling pathways (37). CD4^+^ T cells also indirectly increase neuronal apoptosis by microglia (28). CD4^+^ T cells induce M1 phenotypic polarization by the NF-κB signaling pathway and promote neuronal death. In turn, neurons paradoxically induce the polarization of microglia toward the M1 type, which causes a cascade response and impaires the poststroke outcomes.

Brain-infiltrating CD4^+^ T cells release Nox2-derived superoxide following stroke, which promotes neuronal death (38). Additionally, treatment with CD4^+^ T-cell-derived IL-21 upregulates the expression of the autophagy-associated gene ATG6 in MCAO model mice, which indicates that IL-21 increases neuronal autophagy following IS (39).

### 4.2 CD4^+^ T cells exacerbate cognitive dysfunction in late IS

During the chronic phase of IS, CD4^+^ T cells impair neurological recovery by inducing apoptosis in neural stem/progenitor cells (NSPCs) (11) and initiating a delayed immune response (40).

NSPCs are crucial for late-stage IS repair. Depleting CD4^+^ T cells increases the survival and proliferation of NSPCs in mice at 3 days and 7 days after MCAO. Compared with CD4^+^ T-cell-deficient mice, wild-type mice present higher Fas expression in NSPCs. These findings suggest that CD4^+^ T cells promote NSPC apoptosis after MCAO through the activation of the Fas/FasL pathway (11).

The B-cell response is associated with poststroke cognitive impairment (PSCI). The infiltration and aggregation of B cells in the infarct region are promoted by CD4^+^ T cells. Myeloid cells subsequently surround the infarct and form ectopic lymphoid structures. This process drives B cells to produce antigen-specific autoantibodies, exacerbating late neurological deficits and cognitive decline (40).

## 5 Effects of Th1 cells on different stages of IS

### 5.1 Th1 cells disrupt the BBB and promote the inflammatory response in early IS

The number of tight junctions (TJs) is reduced by Th1-derived IFN-γ following IS, leading to a disruption in BBB integrity (41). This process not only severely damages the BBB but also allows large amounts of IFN-γ to enter the brain, which in turn exacerbates ischemic damage.

Although not verified in models of cerebral ischemia, the inflammatory response is aggravated by Th1 cells through the secretion of proinflammatory cytokines that stimulate immune cell activation and promote proinflammatory cell differentiation (42).

### 5.2 Th1 cells promote neurological functional recovery in late IS

Fourteen days after MCAO, IFN-γ activates the JAK/STAT1 signaling pathway by binding to IFN-γ receptor 1, which is expressed on extracellular vesicles derived from NSCs (43). This process increases neuronal survival and improves poststroke neurological function.

Additionally, IFN-γ can induce the expression of specific miRNAs in exosomes derived from human NSCs to promote neuronal survival (44).

## 6 Effects of Th2 cells on different stages of IS

### 6.1 Th2 cells promote neuronal survival in early IS

Th2 cells exert poststroke protection by secreting cytokines, such as IL-4 and IL-13 (45, 46). The M2 phenotype in microglia/macrophages can be induced by both IL-4 and IL-13. This phenotypic switch alleviates early inflammation in the brain and reduces neuronal death after IS.

In addition, IL-4 potentiates the AKT/MAPK pathway by binding to IL-4R on neurons, which increases neuronal survival (47). However, Liu et al. indicates that IL-4 protects neurons by delaying neuronal apoptosis rather than reducing neuronal loss (48).

### 6.2 Th2 cells promote brain repair in late IS

Treatment with IL-4 and IL-13 promotes white matter recovery and improved sensory and motor functions in the late stage of MCAO (46, 48). IL-4 can directly stimulate oligodendrocyte progenitor cell (OPC) differentiation and remyelination by activating the IL-4/PPARγ signaling pathway, which increases the integrity of the white matter following experimental IS (49).

## 7 Effects of Th17 cells on different stages of IS

### 7.1 Effects of Th17 cells on early IS

#### 7.1.1 Th17 cells disrupt the BBB in early IS

Th17 cells are involved in IS primarily through IL-17 secretion, although γδT cells and astrocytes are the main contributors of IL-17 in early stroke (50).

IL-17A reduces the levels of TJs proteins and increases MMP-2 expression through the interaction with IL-17A receptors on the vascular endothelial cells, which exacerbates BBB injury (51). The binding of IL-17A and IL-17A receptors also activates the myosin light chain and induces the contraction of cerebral microvascular endothelial cells, which increases the interendothelial cell distance and BBB permeability (52).

In addition, IL-17 can directly induce endothelial cell apoptosis through the activation of caspase-3, thereby promoting BBB damage (53).

#### 7.1.2 Th17 cells induce neuronal apoptosis in early IS

The expression of IL-17A receptors on the neuronal surface also increases after IS. The binding of IL-17 and IL-17A receptors can directly induce neuronal death after oxygen–glucose deprivation/recovery (OGD/R) (54). Additionally, IL-17A increases calcineurin-mediated dephosphorylation of the mammalian target of rapamycin (55). It induces excessive neuronal autophagy, which promotes neuronal death. After IS, TRPC6/CREB maintain neuronal survival and function by increasing neuronal cellular tolerance to hypoxia (56). IL-17A can increase TRPC6 hydrolysis to promote neuronal death and neurological dysfunction.

### 7.2 Effects of Th17 cells on late IS

#### 7.2.1 Th17 cells modulate neurogenesis in late IS

The effects of IL-17 and Th17 cells on poststroke neurogenesis continues to be debated.

IL-17 knockout promotes hippocampal neurogenesis and improves cognitive deficits by upregulating the Wnt signaling pathway (57). However, IL-17 derived from astrocytes inhibits calpain 1 activity through p38 MAPK activation, which increases the proliferation and differentiation of NPCs after IS (58). In addition, IL-17 induces neurite outgrowth in the postsympathetic ganglion through the activation of NF-κB (59). These findings suggest that IL-17 from different sources plays different roles in neurogenesis after IS. Therefore, clarifying the source of IL-17 and the effects of different sources of IL-17 on IS is crucial.

Moreover, Th17-derived IL-17 inhibits OPC proliferation and promotes myelin damage by attenuating serine/threonine kinase activity (60). Th17 cells can also directly activate NOTCH1 in OPCs to promote defective remyelination (33). However, researchers have not clearly determined whether Th17 cells affect remyelination after IS.

#### 7.2.2 Th17 cells increase angiogenesis in late IS

Angiogenesis is important for neurologic recovery after IS. Although few studies have investigated the role of IL-17-mediated angiogenesis in IS, IL-17 promotes angiogenesis in individuals with diabetes and inflammatory diseases (61, 62).

In IL-17 knockout mice, the levels of vascular endothelial growth factor and CD34 are reduced in the brain 28 days after MCAO (34). Astrocyte-derived IL-17A reportedly mediates angiogenesis in rats with IS (63), but the effect of Th17 cells on angiogenesis after IS remains to be explored.

## 8 Effects of Tregs on different stages of IS

### 8.1 Tregs mediate both inflammation and thrombosis in AIS

The effect of Tregs on AIS remains paradoxical. Tregs leads to a reduction in infarct volume and an improvement in neurological function by suppressing proinflammatory responses in MCAO mice (35).

**Figure 4:**
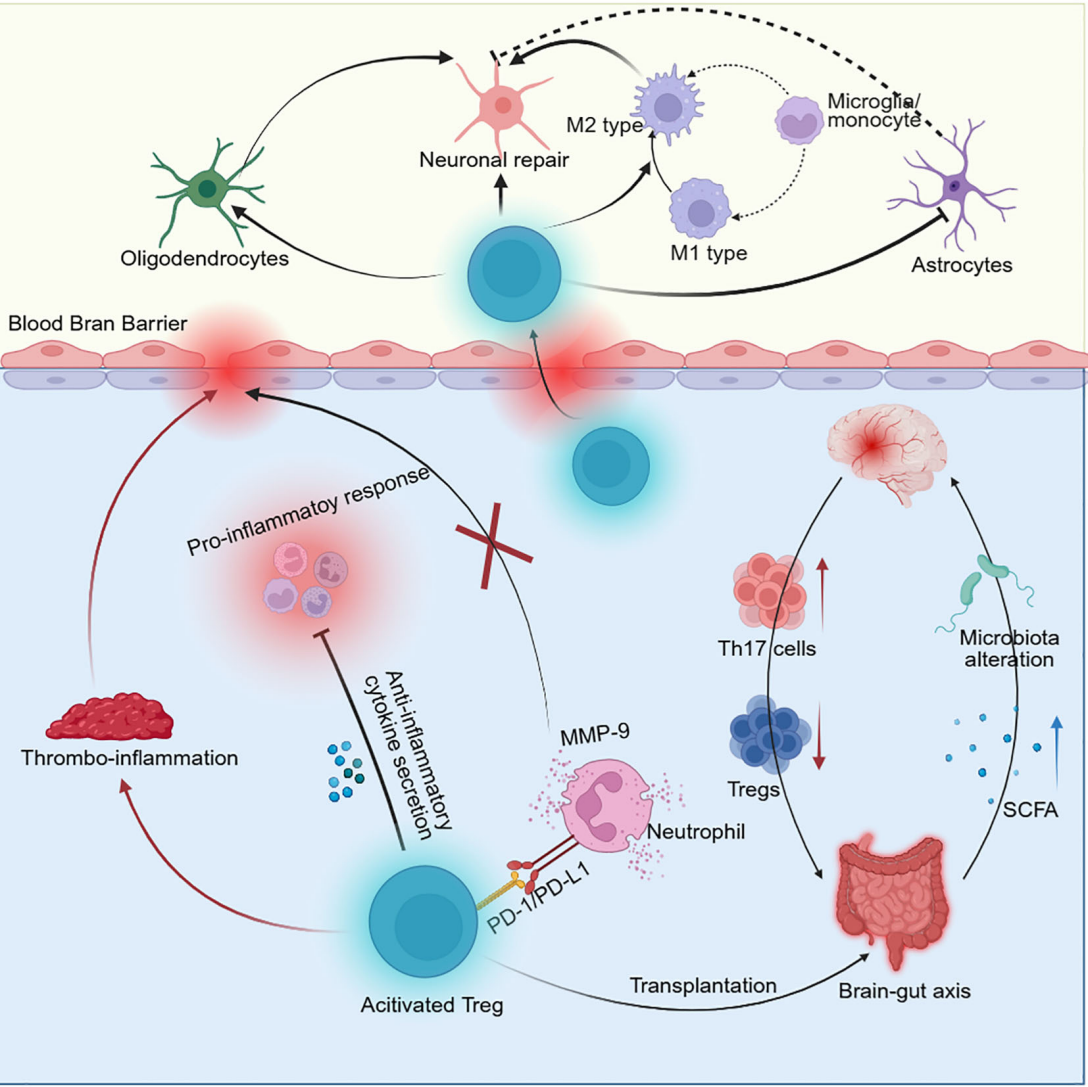
The mechanism by which Treg cells (Tregs) affect ischemic stroke (IS). Tregs inhibit the proinflammatory response in AIS. However, these cells can also exacerbate brain injury through thrombus formation. During subacute and chronic stage of IS, Tregs have a protective effect on IS. In subacute IS, Tregs suppress the inflammatory response by secreting anti-inflammatory cytokines. These cells also inhibit neutrophils-derived MMP-9 to release by PD-1/PD-L1 signaling pathway, which alleviated the blood-brain-barrier (BBB) disruption. In addition, transplantation of Tregs in the gut alter the microbiota and increase the short-chain fatty acid production, which protect the integrity of BBB by brain-gut axis. After Tregs infiltration into the brain in the chronic stage of IS, Tregs promote the neuronal repair. These cells activate oligodendrocytes and M2 phenotypes but inhibited astrocytes to induce neuronal repair.

However, a Foxp3^+^ Treg depletion unexpectedly reduces the infarct volume and improves neurological function scores 24 hours after MCAO (36). This finding was later confirmed by Schuhmann et al. (64). The harmful effects of Tregs differ from their traditional immunoregulatory functions, which are mediated by an inside-out signaling mechanism. These Tregs are predominantly localized in cerebral blood vessels rather than in the brain parenchyma, which is consistent with the physiological process of IS and the high adhesive capacity of Tregs (64). In AIS, brain tissue damage is driven mainly by localized inflammation and microvascular dysfunction. Twenty-four hours after MCAO, chemokines released from the brain tissue activate intracellular signaling pathways in Tregs, which upregulate the expression of surface receptors. These receptors interact with ligands on endothelial cells and platelets, causing thrombus formation and promoting the secondary brain injury (36) (**Figure 4**).

Therefore, Tregs might either exert a protective effect by suppressing inflammation or exacerbate brain damage through thrombus formation in AIS. These findings suggest that the effect of Tregs on IS pathology might depend on their spatiotemporal distribution and microenvironmental regulatory signals.

### 8.2 Effects of Tregs on subacute IS

#### 8.2.1 Tregs suppress the inflammatory response in subacute IS

The anti-inflammatory effects of Tregs appear in the peripheral nervous system rather than in the brain in the subacute phase. These cells secret anti-inflammatory cytokines after IS (65). These cytokines weaken the inflammatory response by inhibiting proinflammatory cytokine production and inducing inflammatory immune cell death in a cytolytic manner.

In addition, peripheral Tregs alleviate the inflammatory response in the brain by the brain–gut axis. An elevated proportion of Tregs in the lamina propria of the large intestine alters the gut microbiota and increases short-chain fatty acid levels (66). Moreover, an increase in the number of Tregs inhibits Th17-cell differentiation and γδT-cell proliferation to disrupt their invasion into the brain (67).

#### 8.2.2 Tregs promote BBB repair in subacute IS

Following IS, endothelial cells activate C-C chemokine receptor type 5 (CCR5) on Tregs, which increases the expression of PD-L1 in Tregs (68). The binding of PD-L1 on Tregs to PD-1 on neutrophils then inhibit MMP-9 production and alleviate BBB disruption.

IL-35 secreted by Tregs also promotes BBB repair (69). Ischemia induces the production of reactive oxygen species (ROS) and thioredoxin-interacting protein (TXNIP) in endothelial cells to exacerbate inflammatory injury. IL-35 inhibits the ROS/TXNIP/caspase-1 pathway and increases TJ expression, thereby attenuating the BBB disruption after IS.

### 8.3 Tregs promote neuronal repair in chronic IS

During the late stage of IS, Treg depletion exacerbates white matter lesions and sensorimotor dysfunction (70).

Tregs induce microglial differentiation toward a reparative phenotype and promote oligodendrocyte regeneration, which leads to improvements in neuronal repair and behavioral functions (24). Tregs also inhibit astrocyte proliferation and neuronal apoptosis through the IL-6/STAT3 pathway, which maintains brain integrity and attenuates poststroke neurological deficits (10).

## 9 Effects of other CD4^+^ T-cell subsets on different stages of IS

### 9.1 The protective effect of Th22 cells on early IS

Although no studies on the poststroke effects of Th22 cells have been published, IL-22, a product of Th22 cells, has been investigated in IS (71).

During the early stage of ischemia, JAK2/STAT3 signaling mediates neuronal apoptosis, oxidative stress and the inflammatory response. IL-22 administration regulates this process to exert a protective effect on early IS.

### 9.2 Tfh cells induce neuronal apoptosis in early IS

In early IS, the harmful effect of Tfh cells was mediated by the secretion of IL-21. IL-21 secreted by Tfh cells activates the JAK/STAT3 pathway by binding to IL-21R expressed on neurons, leading to caspase 3/7-mediated neuronal apoptosis after IS (30).

### 9.3 CD4^+^CD28^-^ T cells increase proinflammatory cytokine release in early IS

CD4^+^CD28^-^ T cells are sometimes classified as specific Th1 cells, which are associated with aging and immune disorders (72). However, CD4^+^CD28^-^ T cells are found only in elderly individuals and patients, which limits the study of these cells (73).

In AIS patients, the number of CD4^+^CD28^-^ T cells in the blood is positively related to the National Institute of Health Stroke Scale (NIHSS) score (74). AIS might activate the costimulatory receptor CD137 on CD4^+^CD28^-^ T cells to induce proinflammatory cytokine release (75).

## 10 Potential of CD4^+^ T cells and their subsets in the clinical diagnosis and treatment of IS

### 10.1 CD4^+^ T cells and their subsets predict the clinical prognosis of IS patients

An early assessment of CD4^+^ T-cell proportions or specific markers in the peripheral blood of AIS patients can aid in predicting the early severity of injury and long-term potential for recovery. Although the association between CD4^+^ T cells and the IS prognosis should theoretically correspond to the effects of their subsets on disease progression, clinical studies have not identified statistically significant correlations for certain CD4^+^ T-cell subsets. Th17 cells and Tregs exhibit the highest predictive value among these subsets, which is likely due to their critical roles throughout IS progression.

#### 10.1.1 Assessment of early brain injury in IS patients: improving diagnostic efficiency and reducing healthcare costs

In patient with early IS, the proportions of CD4^+^ T cells and their subsets in the peripheral blood serve as indicators of the severity of acute brain injury through their correlations with the infarct volume and the NIHSS score.

Compared with healthy individuals, AIS patients presented a lower Treg proportion in the periphery at admission. A reduced Treg proportion was associated with an increased infarct volume and worsened early neurological deficits (76). Further research reported that a greater proportion of CCR5^+^ Tregs was linked to smaller infarct volumes in patients with mild IS than in those with severe IS (77).

Additionally, markers associated with Th17 cell levels provided insights into stroke severity in the early stages. The expression of long noncoding RNA urothelial carcinoma-associated 1 (lncRNA UCA1) was positively correlated with the proportion of Th17 cells in blood samples collected within 12 hours of stroke onset. The significantly elevated levels of the lncRNA UCA1 indicated a short-term functional impairment (78). Conversely, the expression of cell division cycle 42 (CDC42) was negatively correlated with Th17 cell proportions and was significantly lower in AIS patients than in controls. CDC42 expression was negatively correlated with the NIHSS score (79).

These biomarkers enable a rapid assessment of injury severity following IS. Moreover, low-cost blood tests provide an effective preliminary evaluation of a patient’s condition, ultimately reducing treatment costs.

#### 10.1.2 Prediction of the long-term functional recovery in IS patients: optimizing personalized treatment strategies and enhancing long-term disease management

The levels of peripheral CD4^+^ T cells and their subsets not only reflect acute damage but also serve as predictive markers for the prolonged survival and quality of life for patients after IS.

The CD4^+^ T-cell count in the peripheral blood was identified as an independent predictor of the long-term cognitive recovery of AIS patients, but a statistically significant correlation was observed only when it was measured within 1 to 3 days after AIS onset (80).

Many studies have reported that a reduced proportion of Tregs is associated with impaired long-term neurological recovery (76). Further analysis of Treg subsets in relation to stroke prognosis showed that a significant reduction in the naive Treg/memory Treg ratio was associated with cognitive impairment within 90 days after IS (81). In addition, an increased proportion of CCR5^+^ Tregs in the periphery was linked to better functional recovery three months after IS (77).

Among patients admitted within 24 hours of stroke onset, Th17 cell levels on day 7 poststroke showed the strongest positive correlation with cognitive impairment, whereas Th17 levels at all acute-phase time points were positively correlated with IS recurrence and mortality (23). The expression levels of specific genes associated with Th17 cell proportions were also closely related to the IS prognosis. The expression of MALT1, ACC1, and the lncRNA UCA1 was positively correlated with the number of peripheral Th17 cells. Notably, high expression of MALT1 and ACC1 was linked to reduced recurrence-free survival (RFS) (26, 82). Moreover, high lncRNA UCA1 expression not only shortened RFS but also increased the risk of recurrence and mortality within three years (78). In contrast, CDC42 expression negatively correlated with Th17 cell proportions. A decrease in CDC42 expression on day 3 postadmission was associated with an increased risk of recurrence, whereas low expression of CDC42 on day 7 predicted increased mortality (79).

Thus, the early detection of CD4^+^ T-cell proportions and their subsets enables effective prediction of the long-term functional recovery, RFS, and survival outcomes of AIS patients. This predictive capability facilitates the optimization of individualized treatment strategies through early rehabilitation and targeted IS therapy.

### 10.2 Potential of targeting CD4^+^ T cells and their subsets for IS therapy

#### 10.2.1 Blockade of CD4^+^ T-cell migration

##### 10.2.1.1 Monoclonal antibodies

In the past, the relationship between CD4^+^ T cells and IS was poorly understood. Early research suggested that CD4^+^ T-cell infiltration into the brain exacerbated IS, which led to the hypothesis that blocking the migration of CD4^+^ T cells to the brain could improve IS outcomes. Enlimomab, a murine-derived monoclonal antibody that targets CD54, was developed to test this approach (83). Although enlimomab reduced the infiltration of peripheral immune cells and the cerebral infarct volume in animal models, it worsened IS outcomes in clinical trials (**Table 3**). This discrepancy might result from allergic reactions induced by heterologous immunoglobulins.

**Table 3:** The potential of CD4^+^ T cells and their subsets for IS therapy.

Agent	Trial phase	Efficacy	Mechanisms	Reference
Enlimomab	Phase III clinical trial	Enlimomab worsened IS outcomes	Reducing infiltration of peripheral immune cells by the bind of CD54	(83)
Natalizumab	Phase II clinical trial (ACTION)	Drug safety was verified but failed to show therapeutic efficacy	Inhibiting infiltration of peripheral immune cells by targeting α-4 integrin	(87, 88)
Fingolimod	Phase II clinical trial	Drug safety was verified; Improving neurological function both administration alone and combination with revascularization	Blocking lymphocyte migration by inhibiting S1PR activity	(92–95)
CircSCMH1	Preclinical trial	Improving functional recovery in rodent and primate models	Inhibiting infiltration of peripheral immune cells	(97)
Maraviroc	Phase II clinical trial (MARCH)	Improving long-term cognitive recovery	Promoting the recruitment of Tregs into the brain	(103)
JPI-289	Phase II clinical trial	Drug safety was verified and the efficacy evaluation is underway	Increasing peripheral Treg proportion and related anti-inflammatory factors	(14, 104)
Resveratrol	Phase II clinical trial	Improving IS outcomes	Increasing the proportion of Tregs and cytokines	(15)

As a result, a humanized antibody called natalizumab was developed. It inhibited the adhesion and infiltration of CD4^+^ T cells by targeting α-4 integrin (84). However, the therapeutic effects of natalizumab on animal models were inconsistent (85, 86). A phase II clinical trial (ACTION) reported that administering natalizumab (300 mg) intravenously within 9 hours of AIS onset verified the safety of the drug but the researchers failed to show therapeutic efficacy (87). The investigators added groups with different treatment time windows (within 9 hours or 9–24 hours after onset) and different natalizumab doses (300 mg or 600 mg) to verify the efficacy of natalizumab but still did not observe significant differences in efficacy (88). The lack of therapeutic effects was probably due to the inadequate and short-lived blockade of CD4^+^ T-cell infiltration (89). Therefore, simply inhibiting the infiltration of peripheral immune cells into the lesion area appeared insufficient to benefit IS patients.

##### 10.2.1.2 Fingolimod

Fingolimod, an S1P receptor (S1PR) antagonist, was originally approved by the FDA for the treatment of multiple sclerosis (90). After activation through phosphorylation, fingolimod induced the loss of S1PR activity by binding to S1PR on the surface of CD4^+^ T cells. This interaction led to the sequestration of CD4^+^ T cells, which prevented the migration of CD4^+^ T cells to the brain.

In MCAO rats, fingolimod administration reduced infarct volume and alleviated neurological deficits (91). Fu et al. reported that the oral administration of fingolimod (0.5 mg daily; 3 consecutive days) to patients beyond the therapeutic window for alteplase (t-PA) (after 4.5 hours onset) reduced the infarct volume and improved neurological function 7 days after IS (92). In patients eligible for t-PA (within 4.5 hours after onset), the combination of oral fingolimod resulted in a smaller infarct volume and better neurological recovery than t-PA alone (93). In addition, oral fingolimod increased the efficacy of delayed t-PA (after 4.5–6 hours onset) (94). Bridging therapy is also a common approach for revascularization in IS treatment. Compared with patients treated with bridging therapy alone, 80% of patients treated with fingolimod in combination with bridging therapy presented a greater ischemic hemidiaphragm tissue salvage index (95). These findings suggested that fingolimod not only expanded the applicability of IS therapy, but also enhanced the neuroprotective effects of revascularization.

No studies reported severe adverse events including myocardial infarction, recurrent stroke, or liver dysfunction, in AIS patients treated with fingolimod (93). However, fingolimod-induced lymphopenia might increase the risk of infection. Clinical trials reported that these infections were generally mild and could be effectively controlled with short-term antibiotic treatment (92). A novel drug delivery system incorporating cellular manganese dioxide nanospheres loaded with fingolimod was developed. This system not only increased targeted drug delivery to infarct regions but also released oxygen and reduced oxidative stress in addition to the original actions of fingolimod (96).

##### 10.2.1.3 CircSCMH1

CircSCMH1 promoted functional recovery after IS by reducing peripheral immune cell infiltration and upregulating neuroplasticity-related gene expression. However, the clinical application of conventional circSCMH1 delivery systems was limited by safety concerns.

Yang et al. developed engineered exosomes to selectively deliver circSCMH1 to the brain following cerebral ischemia in rodents and primates. This novel delivery approach not only increased the therapeutic efficacy but also extended the treatment window for IS (97).

#### 10.2.2 Regulation of CD4^+^ T-cell differentiation

Recominant IL-33, IL-2/IL-2Ab and IL-4 nanoparticles, which regulated CD4^+^ T-cell differentiation in early IS, exerted neuroprotective effects on IS in animal models (49, 98, 99). These agents decreased the ratio of Th1 cells to Th17 cells while increasing the ratio of Th2 cells to Tregs. Gut microbial therapy might also affect IS outcomes by modulating the migration of specific CD4^+^ T-cell subsets to the lesion area (100).

Rodents and humans differ significantly in their physiological structures and immune systems. Some studies have explored the potential role of humanized cells in IS treatment to simulate the human environment and facilitate the translation of research findings. For example, the transplantation of human pluripotent stem cell-derived vascular endothelial cells promoted the recruitment of Tregs to the injury site, which significantly suppressed neuroinflammation and promoted myelin regeneration after white matter ischemia (101). Additionally, small extracellular vesicles derived from embryonic stem cells could activate the TGF-β/Smad signaling pathway following MCAO, which increased Treg proliferation and ameliorated neurological deficits (102). However, these studies remain in the preclinical trial phase.

##### 10.2.2.1 Maraviroc

Maraviroc, a CCR5 antagonist, was found to improve PSCI after IS. A phase II clinical trial (MARCH) reported that oral maraviroc (300 mg daily or 600 mg daily; 14 consecutive days) improved cognitive recovery in patients who suffered an IS for at least 1 month. Recurrent cardiovascular events and other maraviroc-related adverse events were recorded, but no drug-related adverse reactions were identified (13). The mechanism underlying this effect might be that maraviroc inhibited the proinflammatory response and promoted the recruitment of Tregs into the infarct site (103).

##### 10.2.2.2 JPI-289

The PARP-1 inhibitor JPI-289 was initially reported to protect neuronal survival by attenuating ATP depletion in neurons after OGD (104). JPI-289 also modulated the poststroke immune response by increasing the ratio of peripheral Tregs and related anti-inflammatory factors in IS patients A phase I clinical trial of JPI-289 in Korea reported its safety and tolerability (14). A phase II clinical trial is underway to assess the efficacy of JPI-289.

##### 10.2.2.3 Resveratrol (RV)

Mice pretreated with vitamin D3 had an increased proportion of Tregs and a decreased proportion of Th17 cells in the brain 1 day after MCAO (105). Concurrently, the proportions of Tregs and cytokines were increased centrally and peripherally in rats pretreated with RV (15). The administration of atorvastatin after MCAO increased the proportion of central Tregs and decreased the proportion of peripheral Tregs in rats (106). All these treatments prevented lesion expansion and ameliorated neurological deficits. However, only RV produced clinical benefits in IS patients in clinical trials. Combined treatment with RV and recombinant tissue plasminogen activator (rt-PA) improved the NIHSS score compared to treatment with rt-PA alone. The beneficial effect of RV was more pronounced on patients who received delayed rt-PA treatment (2–4 hours after onset) (107).

## 11 Conclusions and perspectives

This review describes the comprehensive involvement of CD4^+^ T cells and their subsets in the response to IS. We also summarize their effects on different stages of IS and highlight potential clinical strategies for targeted therapies.

The dynamic changes in the immune microenvironment following IS regulate the spatiotemporal distribution of CD4^+^ T cells and their subsets, which further influences the disease progression. In general, elevated levels of CD4^+^ T cells and their subsets during the early stage of IS exacerbate the injury, whereas high numbers of these cells in late IS promote recovery. However, certain exceptions have been noted. Numerous studies have reported that CD4^+^ T cells have detrimental effects on both the early and late stages of IS (37, 40). Unlike the antigen-independent response of CD4^+^ T cells during early IS, the adaptive immune response of CD4^+^ T cells in late stages requires 3 to 7 days to activate (7). Signals released following AIS activate CD4^+^ T cells through antigen presentation, which subsequently recruit peripheral immune cells to infiltrate the brain parenchyma (40). In addition, the CD4^+^ T-cell-mediated cytotoxicity of NSPCs following MCAO contributes to delayed poststroke damage, as NSPCs play a critical role in tissue repair during the late stage of IS (11). Previous studies have often broadly discussed the role of CD4^+^ T cells in late IS. However, significant heterogeneity in their functions in the late stages of IS has been observed with the identification of different CD4^+^ T-cell subsets in recent years. Therefore, studying CD4^+^ T cells as a whole may overlook subset-specific effects, which might lead to biased results. Future research should focus on the specific effects of CD4^+^ T-cell subsets rather than generalizing the effects of total CD4^+^ T cells. Additionally, the dual role of Tregs in AIS might stem from their involvement in multiple pathological mechanisms, including the suppression of inflammation and the disruption of cerebrovascular endothelial integrity (35, 36). Notably, Tregs that mediate different mechanisms exhibit distinct spatial distributions, which suggests the existence of functionally specialized Treg subsets in IS pathology (64). This hypothesis was further supported by the value of Tregs in predicting the IS prognosis. Studies have shown inconsistent correlations between different Treg subsets and IS outcomes. Future research should focus on elucidating the complex mechanisms of Tregs in IS and exploring the functional diversity of their subsets. Evidence from animal models has indicated that Th17 cells contribute to early brain injury following IS (51). Clinical studies also reported that an elevated proportion of Th17 cells was correlated with increased acute damage and worsened long-term prognosis (23). However, Th17 cells remained detectable in the brain 30 days after IS (108). Moreover, Th17 cells mediate neuroregeneration and angiogenesis in other disease models (33, 61). These findings suggest that Th17 cells might have distinct functions in late IS compared with early IS. Therefore, further research is needed to clarify the specific mechanisms underlying the effects of Th17 cells on the late stage of IS. Furthermore, existing studies have focused primarily on the roles of CD4^+^ T cells and their subsets in the brain while neglecting their effects on the periphery. Only by clarifying both the central and peripheral effects of CD4^+^ T cells and their subsets can we selectively target these cells for IS therapy.

IS therapies targeting CD4^+^ T cells remain in the stages of preclinical research and clinical trials. Researchers are actively developing multitarget therapeutic agents and designing nanomaterial-based delivery systems to increase the efficacy and feasibility of CD4^+^ T-cell regulation in IS treatment. However, these studies are still in the preclinical phase, and the number of related investigations remains limited (96, 97). Three drugs exert potential protective effects at different stages of IS. The early administration of fingolimod mitigates brain injury (93). RV injection improves the prognosis of AIS patients undergoing delayed reperfusion therapy (107). Maraviroc treatment contributes to long-term cognitive recovery (13). The potential reasons for the failure of clinical translation described below (1). Preclinical studies generally utilize young male mice, whereas IS primarily affects elderly females with comorbidities. This discrepancy may lead to inconsistent translational outcomes. For example, enlimomab reduced the infarct volume in mice following MCAO. However, clinical trials failed to replicate these benefits (83). In contrast, fingolimod did not improve acute-phase outcomes in MCAO mice but significantly reduced the infarct volume and neurological deficits in AIS patients across multiple clinical trials (109). Future studies should incorporate age- and sex-stratified animal groups or develop models that include metabolic disorders to better align the animal models with the human conditions. Additionally, humanized CD4^+^ T cells and their subsets could be introduced into animal models to elucidate their specific roles in IS pathophysiology (2). A major challenge in IS drug development is the need for a broad therapeutic window to account for the unpredictable onset of IS and variable hospital admission times. Moreover, optimizing the timing of drug administration to balance efficacy and adverse effects remains a critical issue in clinical application (3). Current IS therapies modulate multiple immune cell types but lack specificity in targeting CD4^+^ T-cell subsets. Nonselective treatments may not only reduce the protective effects of certain CD4^+^ T-cell subsets, but also increase the risk of adverse effects due to excessive immune suppression (4). Patients with AIS exhibit a strong inflammatory response in the brain and significant peripheral immunosuppression. A key challenge in clinical translation is developing therapies that exert distinct effects on the central and peripheral immune systems. The development of multitarget drugs in combination with novel biomaterials may provide a promising strategy to overcome the limitations of current IS treatments. A multitarget approach aims to broaden the scope of drug action in multiple pathophysiological processes, thereby addressing the complexity and heterogeneity of IS pathology in different stages. Therefore, targeting CD4^+^ T cells in conjunction with other critical therapeutic targets may not only improve therapeutic efficacy but also extend the treatment window. Furthermore, the integration of multitarget drugs and advanced biomaterials enables targeted delivery and timely release by precise spatiotemporal control. This approach improves treatment precision and optimizes drug efficacy, providing clinically valuable therapeutic strategies for IS patients.

In conclusion, the spatial and temporal distributions and mechanisms of CD4^+^ T cells and their subsets after IS require further investigation. In addition, the development of more targeted therapeutic agents is necessary to expand the scope of IS treatment and increase neuroprotective efficacy.
